# Periodontal Infection and Cardiorespiratory Fitness in Younger Adults: Results from Continuous National Health and Nutrition Examination Survey 1999–2004

**DOI:** 10.1371/journal.pone.0092441

**Published:** 2014-03-24

**Authors:** Ashley Thai, Panos N. Papapanou, David R. Jacobs, Moïse Desvarieux, Ryan T. Demmer

**Affiliations:** 1 Department of Epidemiology, Mailman School of Public Health, Columbia University, New York, New York, United States of America; 2 Division of Periodontics, Section of Oral and Diagnostic Sciences, College of Dental Medicine, Columbia University, New York, New York, United States of America; 3 Division of Epidemiology and Community Health, School of Public Health, University of Minnesota, Minneapolis, Minnesota, United States of America; 4 Department of Nutrition, University of Oslo, Oslo, Norway; 5 Centre de recherche Epidémiologies et Biostatistique, INSERM U1153, Equipe: Méthodes en évaluation thérapeutique des maladies chroniques, Paris, France; University of Toronto, Canada

## Abstract

**Objective:**

Previous studies report associations between periodontal infection and cardiorespiratory fitness but no study has examined the association among younger adults. Our objective was to study the association between clinical measures of periodontal infection and cardiorespiratory fitness levels among a population-based sample of younger adults.

**Methods:**

The Continuous National Health and Nutrition Examination Survey 1999–2004 enrolled 2,863 participants (46% women) who received a partial-mouth periodontal examination and completed a submaximal treadmill test for the assessment of estimated VO_2_ max(eVO_2_ max ). Participants were mean±SD age 33±9 years (range = 20–49 years), 30% Hispanic, 48% White, 19% Black, and 3% other. Mean eVO_2_ max (mL/kg/minute) as well as eVO_2_ max≤32 mL/kg/minute (20th percentile) were regressed across quartiles of mean probing depth and mean attachment loss in multivariable linear and logistic regression models.

**Results:**

After multivariable adjustment, mean eVO_2_ max levels±SE across quartiles of attachment loss were 39.72±0.37, 39.64±0.34, 39.59±0.36, and 39.85±0.39 (P = 0.99). Mean eVO_2_ max±SE across quartiles of probing depth were 39.57±0.32, 39.78±0.38, 39.19±0.25, and 40.37±0.53 (P = 0.28). Similarly, multivariable adjusted mean eVO_2_ max values were similar between healthy participants vs. those with moderate/severe periodontitis: 39.70±0.21 vs. 39.70±0.90 (P = 1.00). The odds ratio (OR) for low eVO_2_ max comparing highest vs. lowest quartile of attachment loss = 0.89[95% CI 0.64–1.24]. The OR for comparing highest vs. lowest probing depth quartile = 0.77[95% CI 0.51–1.15].

**Conclusion:**

Clinical measures of periodontal infection were not related to cardiorespiratory fitness in a sample of generally healthy younger adults.

## Introduction

Healthcare costs in the U.S arising from elevated cardiometabolic risk factors were estimated to be $80 billion in 2005 [1] and a 2012 report by the Centers for Disease Control listed cardiometabolic diseases including hypertension, coronary heart disease, stroke and diabetes among the top seven causes of death in the U.S [2]. Therefore, research that can provide further insight into cardiometabolic disease mechanisms are important because they could inform future prevention efforts and improve population health.

Periodontal infections have been hypothesized as a possible risk factor for cardiometabolic diseases and several studies have reported periodontal disease to be associated with increased risk of incident stroke [3]–[6], coronary heart disease [7], carotid artery atherosclerosis [8]–[10] and type 2 diabetes [11]–[13]. These associations are generally believed to exist independent of traditional risk factors common to both periodontal disease and cardiometabolic disease including low socio-economic status (SES), adverse health behaviors such as smoking, poor diet and reduced physical activity levels. However, the causal nature of these associations remains uncertain and there are currently no results from large-scale randomized controlled trials to formally test the hypothesis.

In order for appropriate intervention trials to be designed, better knowledge is required in regard to plausible biological mechanisms that might connect adverse exposures to the subsequent development of clinical cardiometabolic diseases. In addition, it is important to understand the natural history of associations between periodontal infections and various cardiometabolic outcomes including times during the life course at which associations emerge. This information can help to ensure that susceptible individuals are recruited into studies and that the correct outcomes are studied, thereby maximizing efficiency and allowing for tests of more focused biological hypotheses. Several hypotheses linking infections and cardiometabolic disease have been proposed. For example, chronic low-grade inflammation in response to dysbiotic subgingival microbial communities has been frequently studied in this regard [14]. Periodontal infection/inflammation has also been linked to platelet aggregation, endothelial dysfunction and insulin resistance, all of which are believed to play a pathophysiological role in the development of clinical disease (see [15], [16] for detailed discussion of mechanisms).

Cardiorespiratory fitness is another possible mechanism that might link periodontal infection and cardiometabolic disease although very limited research exists on this topic. Cardiorespiratory fitness is the ability to carry out large muscle, moderate to high intensity exercise over a prolonged period of time and is measured as maximal oxygen consumption (VO_2_ max ) [17], [18] during physical exertion. High cardiorespiratory fitness has been shown to reduce total mortality and cardiovascular disease mortality rates in both men and women [19]–[21] and has also been linked to enhanced insulin sensitivity [22] and reduced diabetes rates [23]. To our knowledge, only three previous studies have reported on the association between periodontal infections and cardiorespiratory fitness and all report increased fitness levels to be associated with better periodontal health [24]–[26]. However, suboptimal periodontal measurement methods were used in two studies, both of which considered periodontal disease as the outcome rather than a predictor of fitness and results were only generalizable to a Japanese population [24], [25]. More recently, Eberhard and colleagues studied the relationship using full-mouth periodontal examinations but the sample was small and restricted to men [26]. There are currently no data available from either younger samples or from a U.S. population.

The purpose of this study was to examine whether or not clinical measures of periodontal infection were related to cardiorespiratory fitness among a large, population-based sample of U.S. adult men and women under age 50 years enrolled in the Continuous National Health and Nutrition Examination Survey (NHANES), 1999–2004.

## Methods

### Study Design and Sample

The Continuous National Health and Nutrition Examination Survey is a continuous annual survey of non-institutionalized United States civilians that uses a stratified multistage probability sampling design [27]. Participants are initially interviewed at home and subsequently invited to attend an in-person examination [27]. 31,126 participants were enrolled in NHANES 1999–2004. The current analyses includes men and women aged 20–49 years who received periodontal examinations, completed a submaximal treadmill exercise test, and for whom information on education, gender, age, smoking status, body mass index (BMI) and hypertension were available (See Figure S1). Individuals were excluded if they were not eligible for the treadmill test due to pregnancy more than 12 weeks (n = 595), physical limitations (n = 792), cardiovascular conditions including congestive heart failure, coronary heart disease or myocardial infarction, angina, stroke, pacemaker or automatic defibrillator, MD instructions for physical activity under MD supervision, chest pain, resting heart rate ≥100 beats/minute, resting systolic blood pressure ≥180 mm Hg, resting diastolic blood pressure ≥100 mm Hg, irregular heart beat (n = 723), asthma (n = 250), self-report lung/breathing problems (n = 515), medications (n = 231), and other (n = 192). They were also excluded if they met Priority 2 stopping criteria due to excessive heart rate (n = 425), excessive blood pressure (n = 20), significant drop in systolic blood pressure (n = 35), variability in heart rate (n = 17), participant request (n = 82), equipment failure (n = 19), participant grips rails (n = 55), participant overexerted (n = 33), technician discretion (n = 32), other (n = 71), cardio fitness test missing (n = 1558), and change in heart rate between the two 3-minute exercises <8 beats/minute so insufficient heart rate to classify fitness (n = 49). Participants were excluded if they were missing information from the oral examination (n = 313), the oral exam was not performed or only partially completed (n = 163), or they were edentulous (n = 11). Individuals were also excluded if education (n = 2), smoking status (n = 4), BMI (n = 6) or hypertension were missing (n = 1). Therefore, n = 2,863 participants are included in the present analysis.

### Oral Examination

Oral examination procedures have been previously described [27]–[29]. Briefly, probing depth and attachment loss measurements were taken by trained, calibrated dentists at the mid-facial and mesio-facial sites in the 1999–2000 cross-section and also at the disto-facial site in the 2001–2002 and 2003–2004 cross-section [27], [28]. The interclass correlation coefficient for probing depth and attachment loss ranged from 0.55–0.87 in the 1999–2000 cross-section, 0.60–0.89 in the 2001–2002 cross-section [27] and 0.61–0.93 in the 2003–2004 cross-section [29].

### Cardiorespiratory Fitness Assessment

Cardiorespiratory fitness assessment has previously been described [30]. Estimated maximal oxygen uptake was predicted from gender, age, BMI and self reported physical activity, and this prediction was used to assign participants to one of eight submaximal treadmill tests to assess cardiorespiratory fitness [18], [30]. The cardiorespiratory fitness exam was conducted by trained health technicians and involved a 2-minute warm-up, two 3-minute exercise stages and a 2-minute cool down with the goal of reaching approximately 75% of the age predicted maximum heart rate by the end of the test [18], [30]. Heart rate was monitored continuously and blood pressure was measured at the end of each stage [18], [30]. Estimated VO_2_ max (eVO_2_ max) was determined based on heart rate measured during the two 3-minute exercise states [31].

### Risk Factor Assessments

Detailed, validated questionnaires were administered by trained NHANES staff to assess various behavioral risk factors as well as disease history. Based on self-report response to questionnaires, the following definitions were created: i) education level was categorized into less than high school, high school, and some college or graduate; ii) race/ethnicity was defined as non-Hispanic White, Hispanic/Mexican, Black and other; iii) smoking status was defined as never, former, and current. Systolic and diastolic blood pressure was measured up to four times using standard methods as recommended by Perloff et al. [32] and the mean of the second and third measurements are used presently. A participant was classified as hypertensive if they reported taking hypertension medication, were ever told they had hypertension, if mean systolic blood pressure was ≥140 mm Hg, or if mean diastolic blood pressure was ≥90 mm Hg. BMI was categorized according to CDC criteria (underweight; ≤18.5, normal; 18.6–24.9, overweight; 25.0–29.9, obese; ≥30) [33]. Metabolic equivalent (MET) minutes per week were calculated as {[(number of times engaged in the activity in past 30 days × average duration of activity × MET score for activity)/30 days] × 7 days}. MET scores were then used to categorize participants as having none, low, moderate or high activity levels using the Physical Activity Guidelines for Americans Summary 2008 (low; ≤499.99, moderate; 500–999.99, high; ≥1000) [34]. Diabetes was defined as glycohemoglobin ≥6.5% or self-report of physician diagnosed diabetes.

### Statistical Analysis

Survey procedures in SAS version 9.2 were used for all analyses. eVO_2_ max was regressed on levels of periodontal disease in crude and multivariable linear and logistic regression models. Periodontal disease was defined based on both mean probing depth and attachment loss in separate regression models. Probing depth and attachment loss were modeled both continuously and categorically in quartiles to enable assessments of dose-responsiveness. The Centers for Disease Control/American Academy of Periodontology (CDC/AAP) Working Group definition was also used to classify participants as having none/mild or moderate/severe periodontitis as previously described [35], [36]. Participants with moderate and severe periodontitis were combined due to a low prevalence of severe periodontitis.

eVO_2_ max was modeled as a continuous outcome in linear regression models (SAS PROC SURVEYREG). The odds of eVO_2_ max≤32 ml/kg/minute (the population-specific 20th percentile of eVO_2_ max) was regressed on levels of the aforementioned periodontal disease categories in logistic regression models using SAS PROC SURVEYLOGISTIC [37]. All p-values presented for linear trend were based on models using a continuous periodontal exposure variable.

Analyses were also conducted in gender subgroups as well as by C-Reactive Protein (CRP) subgroups of >3 mg/L versus CRP≤3 mg/L as adapted from the Centers for Disease Control/American Heart Association statement on inflammatory markers in cardiovascular disease [38]. Subgroup analyses were performed both to reduce possible confounding by gender and inflammation and to assess the evidence for interaction, as previous studies have shown gender [9] and inflammatory [28], [39] interactions with cardiometabolic outcomes.

Based on the NHANES sample size of 2,863 participants available for inclusion in this analysis, the statistical power was 90% to detect a 1.5 mL/kg/minute difference in mean eVO_2_ max levels between the 4^th^ vs. 1^st^ quartile levels of periodontal disease. This assumes a two-sided hypothesis test with α = 0.05 and an eVO_2_ max standard deviation of 9 mL/kg/minute. Mean eVO_2_ max differences of ≥2 mL/kg/minute had ≥99% power. We base these power calculations on prior literature showing that a 3.5 mL/kg/minute is required to increase survival by 12%–17% [40], [41].

## Results

### General Characteristics

Among all participants 46% were women and mean± SD age was 33±8.5 years. Hispanic/Mexican, Black, other, and Non-Hispanic White represented 30%, 19%, 3%, and 48% of the sample, respectively, and 54% reported some level of college education. The majority of participants were never smokers (58%) and 37% had a normal BMI. The percentages of participants reporting, none, low, moderate or high physical activity levels were 31%, 21%, 13% and 35%, respectively. Overall, the mean± SD MET score was 1897±2599 and the mean± SE among participants with low, moderate or high physical activity levels were: 232±6, 719±7, and 3312±95 MET min/week.

### Periodontal Health

Mean±SD attachment loss and probing depth levels were 0.52±0.51 mm and 1.01±0.48 mm. Less than 1% of participants had severe periodontitis, 4% had moderate periodontitis, and <96% were healthy or had mild levels of periodontitis (Table 1). When comparing periodontal status of individuals included in the current analysis to individuals excluded due to ineligibility for the treadmill test (see methods), the mean attachment loss±standard error (SE) values were 0.52±0.01 mm vs. 0.61±0.01 mm (p<0.01) while the mean probing depth±SE values were 1.01±0.01 and 1.08±0.01 (p<0.01) indicating that included participants had better periodontal health. Nevertheless, periodontal status was generally associated with an adverse cardiometabolic risk profile as reported previously in these NHANES 1999–2004 [28] (Table S1).

**Table 1:** Subject Characteristics NHANES 1999–2004 (n = 2863).

	*mean± sd or %*	*n*	min	max
**Attachment Loss (mm)**	0.52±0.51	2863	0.00	4.69
**Probing Depth (mm)**	1.01±0.48	2863	0.21	4.76
**AAP Periodontal Health**				
Severe	0.31	9		
Moderate	4.05	116		
Healthy	95.63	2738		
**eVO_2_ max (ml/kg/min)**	39.73±9.29	2863	17.99	92.15
**Age (years)**	32.87±8.51	2863	20.00	49.00
**Ethnicity**				
Other	2.72	78		
Hispanic or Mexican	30.18	864		
Black	18.97	543		
Non Hispanic White	48.13	1378		
**Gender**				
Male	53.68	1537		
Female	46.32	1326		
**Education**				
Some college or graduate	53.86	1542		
Highschool	24.52	702		
Less than highschool	21.62	619		
**Smoking status**				
Current	26.58	761		
Former	15.61	447		
Never	57.81	1655		
**Poverty-income ratio**	2.80±1.62	2664	0.00	5.00
**BMI**				
Obese	27.00	773		
Overweight	33.64	963		
Underweight	1.99	57		
Normal	37.37	1070		
**MET min/week overall**	1896.80±2598.66	2863		
High	3311.60±3013.64	1010		
Moderate	719.22±141.23	382		
Low	231.65±137.96	588		
None	–	883		
**Hypertension**				
Yes	13.34	382		
No	86.66	2481		
**Diabetes Status**				
Yes	2.41	69		
No	97.59	2794		
**Cholesterol (mg/dL)**	191.70±39.39	2761	87.00	426.00
**Pulse (30 sec ×2 )**	71.23±10.60	2863	44.00	100.00
**WBC count (× 10^9^ cells/L)**	7.15±2.05	2788	2.30	19.50
**CRP (mg/dL)**	0.34±0.69	2772	0.01	16.50

### Cardiorespiratory Fitness

Mean±SD eVO_2_ max was 39.73±9.3 mL/kg/minute and ranged from 17.99 to 92.15 mL/kg/min (Table 1). As previously reported [30], several factors were associated with reduced eVO_2_ max such as older age, Black or other ethnicity, female gender, high school education, former and never smoking status, overweight/obesity, lower physical activity levels, hypertension, diabetes, and elevated cholesterol, pulse, white blood cell count and CRP (Tables S2 and S3).

### Association between Periodontal Health & Cardiorespiratory fitness

Unadjusted mean levels of estimated maximal oxygen uptake across quartiles of attachment loss and probing depth are shown in Table S1. After multivariable adjustment, mean eVO_2_ max (mL/kg/minute)±SE across quartiles of attachment loss were 39.72±0.37, 39.64±0.34, 39.59±0.36, and 39.85±0.39 (P = 0.99, Table 2). Mean eVO_2_ max (mL/kg/minute) ± SE across quartiles of probing depth were 39.57±0.32, 39.78±0.38, 39.19±0.25, and 40.37±0.53 (P = 0.28). Similarly, multivariable adjusted mean eVO_2_ max values were similar between healthy participants vs. those with moderate/severe periodontitis: 39.70±0.21 vs. 39.70±0.90 mL/kg/min (P = 1.00).

**Table 2:** Mean eVO_2_ max Across Quartiles of Attachment Loss and Probing Depth, NHANES 1999–2004.

	eVO_2_ (ml/kg/min)				
Attachment Loss	Quartile 1	Quartile 2	Quartile 3	Quartile 4	
	*means±se*	*means±se*	*means±se*	*means±se*	p for trend
**Model 1**	*(n = 735)*	*(n = 695)*	*(n = 703)*	*(n = 730)*	
	39.47±0.39	39.41±0.44	39.32±0.46	40.21±0.48	0.21
**Model 2**	*(n = 735)*	*(n = 695)*	*(n = 703)*	*(n = 730)*	
	39.89±0.34	39.54±0.35	39.47±0.38	39.89±0.47	0.95
**Model 2b**	*(n = 692)*	*(n = 651)*	*(n = 649)*	*(n = 672)*	
	39.69±0.39	39.48±0.35	39.20±0.40	39.78±0.42	0.87
**Model 3**	*(n = 692)*	*(n = 651)*	*(n = 649)*	*(n = 672)*	
	39.70±0.36	39.58±0.36	39.50±0.38	39.90±0.40	0.92
**Model 4**	*(n = 660)*	*(n = 630)*	*(n = 636)*	*(n = 655)*	
	39.72±0.37	39.64±0.34	39.59±0.36	39.85±0.39	0.99
**Model 5**	*(n = 660)*	*(n = 630)*	*(n = 636)*	*(n = 655)*	
	39.70±0.36	39.63±0.34	39.60±0.36	39.86±0.39	0.97
**Probing Depth**	*means±se*	*means±se*	*means±se*	*means±se*	p for trend
**Model 1**	*(n = 718)*	*(n = 735)*	*(n = 700)*	*(n = 710)*	
	39.31±0.33	39.30±0.47	39.17±0.36	40.86±0.60	0.01
**Model 2**	*(n = 718)*	*(n = 735)*	*(n = 700)*	*(n = 710)*	
	39.87±0.34	39.60±0.36	39.02±0.29	40.37±0.59	0.54
**Model 2b**	*(n = 691)*	*(n = 688)*	*(n = 640)*	*(n = 645)*	
	39.75±0.38	39.47±0.36	38.90±0.32	40.07±0.51	0.67
**Model 3**	*(n = 691)*	*(n = 688)*	*(n = 640)*	*(n = 645)*	
	39.57±0.32	39.67±0.36	39.14±0.26	40.41±0.50	0.22
**Model 4**	*(n = 671)*	*(n = 658)*	*(n = 625)*	*(n = 627)*	
	39.57±0.32	39.78±0.38	39.19±0.25	40.37±0.53	0.28
**Model 5**	*(n = 671)*	*(n = 658)*	*(n = 625)*	*(n = 627)*	
	39.55±0.32	39.79±0.38	39.18±0.25	40.38±0.53	0.28

Model 1: crude.

Model 2: adjusted for age, ethnicity, gender, education, smoking.

Model 2b: adjusted for age, ethnicity, gender, education, smoking, poverty-income ratio.

Model 3: Model 2+ BMI and MET.

Model 4: Model 3+ hypertension, diabetes, cholesterol and pulse.

Model 5: Model 4+ CRP, WBC.

In multivariable logistic regression models, participants in the highest quartile of attachment loss had lower odds of reduced eVO_2_ max (≤31.98 mL/kg/minute), though this was not significant: OR = 0.89[95%CI 0.64–1.24], (Table 3). Similarly individuals in the fourth quartile of probing depth vs. the first, had lower odds of reduced eVO_2_ max: OR = 0.77[95%CI 0.51–1.15]. Participants with CDC/AAP defined moderate/severe periodontitis had a statistically significant lower odds of reduced eVO_2_ max: OR = 0.48[95%CI 0.23–0.98] (Table 4).

**Table 3:** Odds Ratios for Low Levels of eVO_2_ max (≤31.98 mL/kg/minute) Across Quartiles of Attachment Loss and Probing Depth, NHANES 1999–2004.

	Model 1 (n = 2761)				Model 2 (n = 2760)			
	*OR*	*95% CI*			*OR*	*95% CI*		
**Attachment Loss**								
Quartile 1	ref	ref		ref	ref	ref		ref
Quartile 2	1.09	0.78	–	1.51	1.09	0.78	–	1.51
Quartile 3	1.23	0.85	–	1.78	1.22	0.84	–	1.77
Quartile 4	0.89	0.64	–	1.24	0.89	0.64	–	1.24
**Age (years)**	1.04	1.02	–	1.06	1.04	1.02	–	1.06
**Ethnicity**								
Other	1.23	0.68	–	2.23	1.23	0.68	–	2.21
Hispanic or Mexican	1.43	0.99	–	2.07	1.41	0.97	–	2.03
Black	2.17	1.62	–	2.91	2.2	1.65	–	2.93
Non Hispanic White	ref	ref		ref	ref	ref		ref
**Gender**								
Male	0.1	0.07	–	0.14	0.1	0.07	–	0.15
Female	ref	ref		ref	ref	ref		ref
**Education**								
Some college or graduate	1.24	0.79	–	1.94	1.23	0.78	–	1.93
Highschool	1.41	1.01	–	1.97	1.41	1	–	1.99
Less than highschool	ref	ref		ref	ref	ref		ref
**Smoking status**								
Current	0.62	0.45	–	0.87	0.61	0.45	–	0.84
Former	1.12	0.78	–	1.61	1.12	0.78	–	1.62
Never	ref	ref		ref	ref	ref		ref
**BMI**								
Obese	1.68	1.21	–	2.34	1.61	1.19	–	2.18
Overweight	1.33	0.95	–	1.86	1.3	0.94	–	1.81
Underweight	0.58	0.24	–	1.39	0.59	0.25	–	1.39
Normal	ref	ref		ref	ref	ref		ref
**MET min/week**								
High	0.57	0.41	–	0.8	0.58	0.42	–	0.8
Moderate	0.8	0.51	–	1.24	0.8	0.51	–	1.26
Low	0.97	0.7	–	1.34	0.97	0.7	–	1.34
None	ref	ref		ref	ref	ref		ref
**Hypertension**								
Yes	0.94	0.65	–	1.36	0.93	0.64	–	1.35
No	ref	ref		ref	ref	ref		ref
**Diabetes Status**								
Yes	1.24	0.58	–	2.68	1.24	0.57	–	2.68
No	ref	ref		ref	ref	ref		ref
**Cholesterol (mg/dL)**	1	1	–	1.01	1	1	–	1.01
**Pulse (30 sec x2 )**	1.04	1.02	–	1.05	1.04	1.02	–	1.05
**WBC count (x 10^9^ cells/L)**					1.01	0.94	–	1.09
**CRP (mg/dL)**					1.07	0.92	–	1.25
							
**Probing Depth**								
Quartile 1	ref	ref		ref	ref	ref		ref
Quartile 2	1.07	0.82	–	1.39	1.07	0.82	–	1.4
Quartile 3	0.94	0.67	–	1.32	0.94	0.67	–	1.32
Quartile 4	0.77	0.51	–	1.15	0.77	0.51	–	1.15
**Age (years)**	1.04	1.02	–	1.05	1.04	1.02		1.06
**Ethnicity**								
Other	1.24	0.68	–	2.25	1.23	0.68	–	2.23
Hispanic or Mexican	1.47	1.01	–	2.14	1.45	1	–	2.11
Black	2.19	1.61	–	2.97	2.22	1.64	–	3
Non Hispanic White	ref	ref		ref	ref	ref		ref
**Gender**								
Male	0.1	0.07	–	0.15	0.1	0.07	–	0.15
Female	ref	ref		ref	ref	ref		ref
**Education**								
Some college or graduate	1.22	0.78	–	1.9	1.21	0.77	–	1.9
Highschool	1.38	0.98	–	1.95	1.38	0.97	–	1.97
Less than highschool	ref	ref		ref	ref	ref		ref
**Smoking status**								
Current	0.63	0.44	–	0.89	0.61	0.44	–	0.85
Former	1.14	0.8	–	1.63	1.14	0.79	–	1.64
Never	ref	ref		ref	ref	ref		ref
**BMI**								
Obese	1.68	1.22	–	2.33	1.61	1.19	–	2.18
Overweight	1.32	0.95	–	1.84	1.3	0.94	–	1.79
Underweight	0.56	0.24	–	1.31	0.56	0.24	–	1.3
Normal	ref	ref		ref	ref	ref		ref
**MET min/week**								
High	0.57	0.41	–	0.8	0.57	0.41	–	0.8
Moderate	0.78	0.5	–	1.22	0.79	0.5	–	1.24
Low	0.97	0.7	–	1.34	0.97	0.7	–	1.35
None	ref	ref		ref	ref	ref		ref
**Hypertension**								
Yes	0.95	0.66	–	1.38	0.94	0.65	–	1.36
No	ref	ref		ref	ref	ref		ref
**Diabetes Status**								
Yes	1.26	0.58	–	2.72	1.26	0.58	–	2.72
No	ref	ref		ref	ref	ref		ref
**Cholesterol (mg/dL)**	1	1	–	1.01	1	1	–	1.01
**Pulse (30 sec x2 )**	1.04	1.02	–	1.05	1.04	1.02	–	1.05
**WBC count (x 10^9^ cells/L)**					1.02	0.95	–	1.09
**CRP (mg/dL)**					1.07	0.92	–	1.24

Model 1: adjusted for age, race/ethnicity, gender, education, smoking, poverty-income ratio, BMI, MET-score, hypertension, diabetes, cholesterol, pulse.

Model 2: Model 1+ C-reactive protein and white blood cell count.

**Table 4:** Odds Ratios for Low eVO_2_ max (≤31.98 mL/kg/minute) Across CDC/AAP Categories of Periodontitis, NHANES 1999–2004 (n = 2,760).

	Model 1 (n = 2761)				Model 2 (n = 2760)			
	*OR*	*95% CI*			*OR*	*95% CI*		
**AAP Periodontal Health**								
Healthy	ref	ref		ref	ref	ref		ref
Moderate/Severe	0.48	0.23	–	0.98	0.48	0.23	–	0.98
**Age (years)**	1.04	1.02	–	1.06	1.04	1.02	–	1.06
**Ethnicity**								
Other	1.24	0.68	–	2.28	1.24	0.68	–	2.27
Hispanic or Mexican	1.43	0.99	–	2.06	1.41	0.98	–	2.02
Black	2.18	1.63	–	2.90	2.20	1.66	–	2.93
Non Hispanic White	ref	ref		ref	ref	ref		ref
**Gender**								
Male	0.10	0.07	–	0.14	0.10	0.07	–	0.15
Female	ref	ref		ref	ref	ref		ref
**Education**								
Some college or graduate	1.22	0.77	–	1.93	1.22	0.77	–	1.93
Highschool	1.38	0.98	–	1.94	1.38	0.97	–	1.97
Less than highschool	ref	ref		ref	ref	ref		ref
**Smoking status**								
Current	0.64	0.45	–	0.91	0.62	0.45	–	0.87
Former	1.15	0.81	–	1.64	1.15	0.81	–	1.65
Never	ref	ref		ref	ref	ref		ref
**BMI**								
Obese	1.66	1.20	–	2.30	1.59	1.18	–	2.15
Overweight	1.32	0.94	–	1.84	1.29	0.93	–	1.79
Underweight	0.57	0.24	–	1.32	0.57	0.25	–	1.32
Normal	ref	ref		ref	ref	ref		ref
**MET min/week**								
High	0.58	0.41	–	0.80	0.58	0.42	–	0.81
Moderate	0.79	0.51	–	1.23	0.80	0.51	–	1.25
Low	0.97	0.70	–	1.34	0.97	0.70	–	1.34
None	ref	ref		ref	ref	ref		ref
**Hypertension**								
Yes	0.95	0.66	–	1.38	0.94	0.65	–	1.37
No	ref	ref		ref	ref	ref		ref
**Diabetes Status**								
Yes	1.26	0.59	–	2.69	1.25	0.58	–	2.69
No	ref	ref		ref	ref	ref		ref
**Cholesterol (mg/dL)**	1.00	1.00	–	1.01	1.00	1.00	–	1.01
**Pulse (30 sec x2 )**	1.04	1.02	–	1.05	1.04	1.02	–	1.05
**WBC count (x 10^9^ cells/L)**					1.01	0.94	–	1.09
**CRP (mg/dL)**					1.07	0.93	–	1.24

Model 1: adjusted for age, race/ethnicity, gender, education, smoking, poverty-income ratio, BMI, MET-score, hypertension, diabetes, cholesterol, pulse.

Model 2: Model 1+ C-reactive protein and white blood cell count.

Stratification by gender in both multivariable linear and logistic regression analyses showed no clinically meaningful or statistically significant variation in the association between either probing depth or attachment loss and eVO_2_ max (Tables S4 & S5). Gender specific analyses for CDC/AAP periodontitis definition were not feasible given the very low prevalence of individuals with moderate/severe periodontitis. Additional analyses according to subgroups of CRP were also null (Tables S6 & S7).

## Discussion

We have examined the association between clinical measures of periodontal infection and levels of eVO_2_ max among a population-based, representative sample of younger adult men and women in the U.S. We report no association between periodontal infection and eVO_2_ max. These results were consistent after multivariable confounder adjustment, using multiple clinical definitions of periodontal infection and upon stratification by subgroups of gender and levels of CRP.

The overall magnitude of association between periodontal measures and eVO_2_ max was very small and clinically insignificant. These results suggest mean differences in eVO_2_ max between participants with highest vs. lowest levels of periodontal disease to be <1.0 mL/kg/minute while previous studies have shown that an increase in eVO_2_ max of at least 3.5 mL/kg/minute is required to increase survival by 12%–17% [40], [41]. The observed statistically significant inverse association between probing depth and eVO_2_ max in crude analyses was likely influenced by the negative confounding related to substantial elevation in eVO_2_ max among men vs. women coupled with higher levels of periodontal disease among men. Similarly, current smokers also had slight elevations in eVO_2_ max and higher levels of periodontal disease. Multivariable adjustment for gender and smoking as well as several other established cardiometabolic risk factors removed any observed associations between periodontal status and eVO_2_ max.

These results might help to advance our understanding of, and increase our confidence in, possible biological mechanisms underlying observed associations between periodontal infections and cardiometabolic risk. Specifically, the current null finding argues against mechanisms related to cardiorespiratory fitness (at least in younger, generally healthy, adult populations) and therefore indirectly supports other mechanisms. For example, the role of host inflammatory response to dysbiotic periodontal biofilms has been hypothesized as a mechanism linking infection and cardiometabolic risk by increasing the risk for insulin resistance [28], atherosclerotic development [8] and/or endothelial dysfunction [42]. This mechanism could produce a parallel decline in eVO_2_ max, leading to associations of periodontal disease and eVO_2_ max in older adults.

Our results do not directly support previous findings regarding clinical periodontal disease and cardiorespiratory fitness. Wakai et al. found that an eVO_2_ max of >30 mL/kg/minute vs. <10 ml/kg/min was associated with significantly improved levels of oral health as assessed by the Community Periodontal Index of Treatment Needs (CPITN) after adjusting for sex, age, smoking, glucose and debris index [24]. Similarly, Shimazaki et al. also reported that the highest quintile of eVO_2_ max was significantly associated with reduced odds of Community Periodontal Index (CPI) defined periodontitis (OR = 0.41, 95%CI = 0.21–0.81). However, the fact that periodontal status was studied as the outcome in both studies prevents the assessment of the variation in eVO_2_ max across increasing severity and extent of periodontal disease to determine the clinical significance of observed associations. As importantly, both previous studies were conducted among a sample of Japanese adults who were approximately 7 years [25] to 20 years [24] older than participants included in the current sample. Therefore, it is possible that the range of periodontal disease and/or eVO_2_ max might have enabled more meaningful analyses in previous studies. Alternatively, it is possible that this sample is simply too young and healthy (in terms of periodontal and cardiorespiratory health) for associations between infectious burden and CVD fitness to have emerged. Accordingly, hypotheses concerning infection and markers of chronic CVD risk markers generally assume that sustained infectious exposure accumulated over the lifecourse is necessary for meaningful risk to accrue [43].

If future studies are designed to examine associations between periodontal infection and cardiorespiratory fitness, these data, coupled with results from previous reports, suggest that older adults (possibly >50 years of age) should be enrolled if the aims are to examine cross-sectional relationships. In the context of longitudinal studies, the lack of association among participants under 50 years could be a strong advantage for future research designs, as it would enable participants to be enrolled prior to the emergence of associations enabling more clarity in regard to temporality of associations. However, planned follow-up times would need to be sufficiently long to enable relationships to emerge.

It is possible that half-mouth periodontal examinations used in the currently analyzed NHANES survey-cycles might have led to misclassification of individuals with periodontitis as being periodontally healthy which could have biased results towards the null. However, other studies from NHANES have demonstrated associations between periodontitis and other cardiometabolic outcomes such as peripheral vascular disease [44], inflammation [45] and insulin resistance [28] using half-mouth exams. Moreover, previous studies have shown that relative measures of attachment loss and probing depth based on half-mouth exams are good correlates of general periodontal health status, including exposure to pathological biofilms [46]–[48]. It is also possible that incidence-prevalence bias might have biased results towards the null or possibly induced subtle inverse associations [49]. This can occur if periodontal infections directly cause conditions that would preclude individuals with reduced eVO_2_ max from participating in the study generally, or in the treadmill test specifically. The inverse association between current smoking status and eVO_2_ max is supportive of this form of bias in the data. Alternatively, the fact that increased BMI, reduced physical activity levels, and older age were associated with lower eVO_2_ max as expected and in a manner consistent with previous reports [30] suggests that the influence of incidence-prevalence bias in these data is quite modest and unlikely to be of a magnitude necessary to induce inverse associations. As eVO_2_ max was estimated for adults aged 20–49 years in NHANES these findings cannot be generalized to adults older than 50 years of age with a range of health conditions used to exclude participants from the treadmill test. The youth and relatively good health of our sample might also explain why our findings are discordant with previous studies.

Although we did not account for occupational activity levels, it is unlikely that this information would meaningfully bias our current analysis. As occupational activities are likely to be strongly correlated with several factors that we did include in our multivariable adjustments (i.e., body composition, blood pressure, educational level and pulse), work related activity level is likely to have been largely accounted for indirectly. Moreover, the lack of adjustment for daily work activity would only result in an inverse association (i.e., periodontitis related to increased eVO_2_ max) it would require that increased activity at work be strongly associated with both periodontitis and increased eVO_2_ max independent of all factors adjusted for in our statistical models.

In summary, we report no association between clinical measures of periodontitis and cardiorespiratory fitness in a large sample of young U.S adults. While the lack of full-mouth periodontal exams and/or the limited range of clinical periodontal disease and eVO_2_ max levels might have biased results towards the null, these data suggest that clinical indicators of periodontal infection are not associated with cardiorespiratory fitness in a clinically meaningful fashion among younger adults.

## Supporting Information

Figure S1 **Subject exclusion flow chart.** Figure shows how authors reached final sample size included in analyses through exclusion of individuals without main exposure and outcome data, or who were missing important variables adjusted for in models.
(TIFF)

Table S1 **Characteristics Across Quartiles of Attachment Loss and Probing Depth (NHANES 1999–2004).**
(XLSX)

Table S2 **eVO_2_ Max Across Age, Physical Activity Level, Gender, Ethnicity and BMI (NHANES 1999–2004).**
(XLSX)

Table S3 **Change in VO_2_ Estimated Across Continuous Attachment Loss and Probing Depth.**
(XLSX)

Table S4 **Change in VO_2_ Estimated Across Quartiles of Attachment Loss and Probing Depth by Gender.**
(XLSX)

Table S5 **OR for Estimated VO_2_≤31.98 (19.94%) Across Quartiles of Attachment Loss and Probing Depth by Gender.**
(XLSX)

Table S6 **Change in VO_2_ Estimated Across Continuous Attachment Loss and Probing Depth by CRP> or ≤.3 mg/dL.**
(XLSX)

Table S7 **Change in VO_2_ Estimated Across Quartiles of Attachment Loss and Probing Depth by CRP.**
(XLSX)
