# Supplementary figures and images for: Periodontal Infection and Cardiorespiratory Fitness in Younger Adults: Results from Continuous National Health and Nutrition Examination Survey 1999–2004
Source: PLoS One. 2014 Mar 24;9(3):e92441. doi: 10.1371/journal.pone.0092441 (PMC3963905; doi:10.1371/journal.pone.0092441)

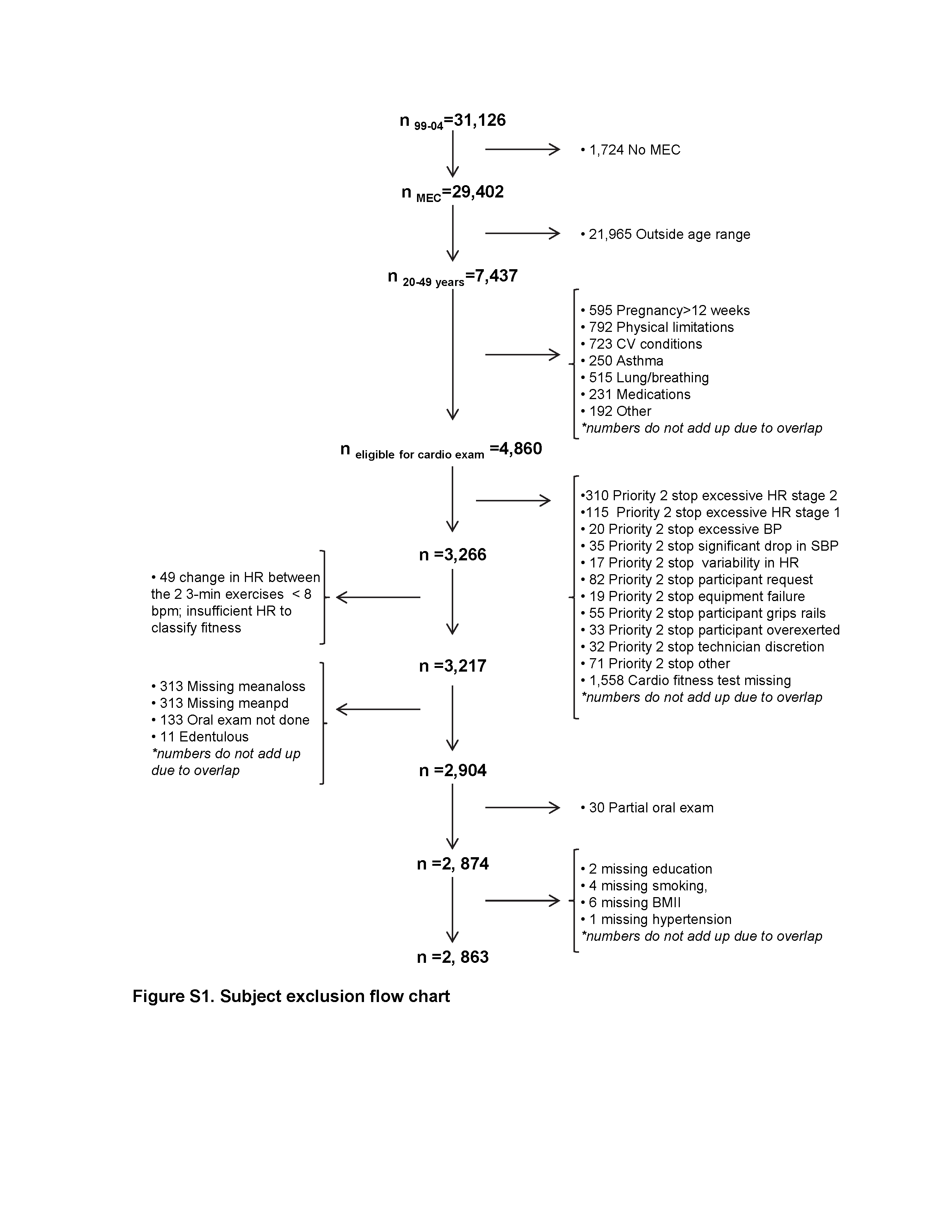

Supplement: Figure S1 — Subject exclusion flow chart. Figure shows how authors reached final sample size included in analyses through exclusion of individuals without main exposure and outcome data, or who were missing important variables adjusted for in models. (TIFF) [file pone.0092441.s001.tif]
